# A New Hadrosauroid Dinosaur from the Early Late Cretaceous of Shanxi Province, China

**DOI:** 10.1371/journal.pone.0077058

**Published:** 2013-10-18

**Authors:** Run-Fu Wang, Hai-Lu You, Shi-Chao Xu, Suo-Zhu Wang, Jian Yi, Li-Juan Xie, Lei Jia, Ya-Xian Li

**Affiliations:** 1 Shanxi Museum of Geological and Mineral Science and Technology, Taiyuan, Shanxi Province, People's Republic of China; 2 Key Laboratory of Vertebrate Evolution and Human Origins of Chinese Academy of Sciences, Institute of Vertebrate Paleontology and Paleoanthropology, Chinese Academy of Sciences, Beijing, People's Republic of China; Royal Ontario Museum, Canada

## Abstract

**Background:**

The origin of hadrosaurid dinosaurs is far from clear, mainly due to the paucity of their early Late Cretaceous close relatives. Compared to numerous Early Cretaceous basal hadrosauroids, which are mainly from Eastern Asia, only six early Late Cretaceous (pre-Campanian) basal hadrosauroids have been found: three from Asia and three from North America.

**Methodology/Principal Findings:**

Here we describe a new hadrosauroid dinosaur, *Yunganglong datongensis* gen. et sp. nov., from the early Late Cretaceous Zhumapu Formation of Shanxi Province in northern China. The new taxon is represented by an associated but disarticulated partial adult skeleton including the caudodorsal part of the skull. Cladistic analysis and comparative studies show that *Yunganglong* represents one of the most basal Late Cretaceous hadrosauroids and is diagnosed by a unique combination of features in its skull and femur.

**Conclusions/Significance:**

The discovery of *Yunganglong* adds another record of basal Hadrosauroidea in the early Late Cretaceous, and helps to elucidate the origin and evolution of Hadrosauridae.

## Introduction

Hadrosauroids were facultative bipedal dinosaurs that dominated Cretaceous Laurasian megaherbivorous niches [1], [2]. During the Late Cretaceous, they gave rise to hadrosaurid dinosaurs, which are characterized by duck-like bills and complex grinding dentitions that rival those of horses and bovids [3]. However, the origin of hadrosaurids is far from clear, mainly due to the paucity of early Late Cretaceous close relatives. Basal hadrosauroids are mainly known from the Early Cretaceous of Eastern Asia, and especially northern China [4]. Until now, only six pre-Campanian Late Cretaceous basal hadrosauroids have been reported: three from Asia (*Shuangmiaosaurus gilmorei*, *Levnesovia transoxiana*, and *Nanyangosaurus zhugeii*, see discussion section below on its age) and three from North America (*Eolambia caroljonesa*, *Protohadros byrdi*, and *Jeyawati rugoculus*) [5]–[10]. Therefore, the discovery of new early Late Cretaceous basal hadrosauroids has important phylogenetic and paleobiogeographical significance and can help elucidate the evolution of hadrosauroids, especially the origin of hadrosaurids.

Definitions of relevant taxa follow Sereno [11]: Hadrosauriformes is the least inclusive clade containing *Iguanodon bernissartensis* Boulenger in Beneden 1881 and *Parasaurolophus walkeri* Parks 1922, Hadrosauroidea is the most inclusive taxon containing *Parasaurolophus walkeri* Parks 1922 but not *Iguanodon bernissartensis* Boulenger in Beneden 1881, and Hadrosauridae is the least inclusive taxon containing *Saurolophus osborni* Brown 1912 and *Parasaurolophus walkeri* Parks 1922 and including *Hadrosaurus foulkii* Leidy 1858. The term “basal hadrosauroids” here refers to all members of non-Hadrosauridae Hadrosauroidea.

**Figure 1:**
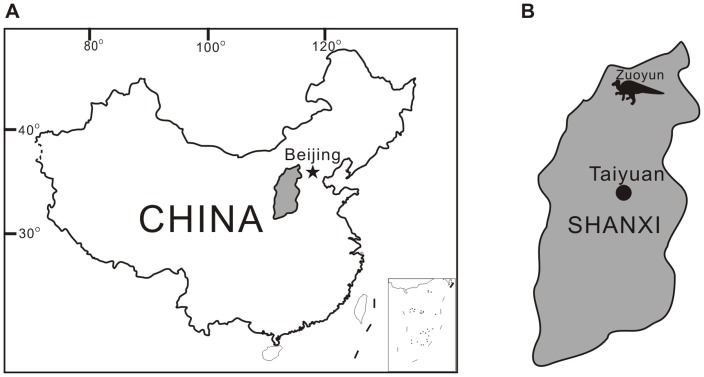
Locality of *Yunganglong datongensis* (SXMG V 00001). (A)-Shanxi Province in China. (B)-Zuoyun County in Shanxi Province.

In 1958, C. C. Young (Z.-J. Yang) [12] reported the first dinosaur record in Shanxi Province of northern China. These remains were recovered from two localities in Zuoyun County of northern Shanxi (Fig. 1). The material is fragmentary, and Young did not name any new dinosaur species; rather, he assigned some of them to existing taxa [12]. The first locality (Xinyaogou) yielded theropod (cf. *Velociraptor mongoliensis*, represented by a single tooth), ceratopsian (cf. *Microceratops gobiensis*, represented by two partial mandibles, three isolated teeth, several vertebrae, one humerus, one femur, and other fragmentary specimens), and hadrosauroid (*Bactrosaurus johnsoni*, represented by two isolated teeth, some vertebrae including a series of 25 caudals, one rib, one right humerus, and several manual and pedal bones). The second locality (Zhanmagou) yielded Sauropoda indet. (represented by one partial dorsal and one fragmentary caudal vertebrae) and Allosauridae indet. (represented by one caudal, one partial right scapula, two distal ends of ischia, one proximal end of right tibia, one partial right fibula, one left metatarsal, and one pedal phalanx). Based on the different dinosaur components, Young considered the Xinyaogou dinosaur assemblage to be Late Cretaceous in age and the Zhanmagou dinosaur assemblage to be Early Cretaceous or earlier in age.

More than half a century later, the Department of Land and Resources of Shanxi Province initiated a project to find dinosaurs for the Shanxi Museum of Geology (SXMG) now being constructed. In 2011 and 2012, a number of dinosaur localities were discovered and excavated in Zuoyun County. Preliminary observations indicate the existence of hadrosauroid, ankylosaur, and ceratopsian remains from the early Late Cretaceous Zhumapu Formation, and stegosaur and sauropod remains from the late Early Cretaceous Zuoyun Formation. In this paper, a partial associated hadrosauroid skeleton from 2011 Locality 7 (SXMG V 00001, field number ZY007) is described and diagnosed as a new genus and species.

## Methods

No permits were required for this study, which complied with all relevant regulations. We obtained permission from the Shanxi Museum of Geological and Mineral Science and Technology to access the collections. The specimens were discovered and excavated by the crew (including co-authors of this study) of the Shanxi Museum of Geological and Mineral Science and Technology.

### Nomenclatural Acts

The electronic edition of this article conforms to the requirements of the amended International Code of Zoological Nomenclature, and hence the new names contained herein are available under that Code from the electronic edition of this article. This published work and the nomenclatural acts it contains have been registered in ZooBank, the online registration system for the ICZN. The ZooBank LSIDs (Life Science Identifiers) can be resolved and the associated information viewed through any standard web browser by appending the LSID to the prefix “http://zoobank.org/”. The LSID for this publication is: urn:lsid:zoobank.org:pub:26AD8279-F3F8-4A6F-A16F-B163BB54F7EE. The electronic edition of this work was published in a journal with an ISSN, and has been archived and is available from the following digital repositories: PubMed Central, LOCKSS.

### Phylogenetic Analysis

Numerous cladistic analyses have been conducted in order to elucidate the phylogenetic relationships among hadrosauroids in recent years; for example, at least four papers were published in 2012 [4], [13]–[15]. However, many of these focused on the phylogeny of Hadrosauridae or its subclades; for example, lambeosaurine was the subject of [13], while saurolophine was the subject of [14]. Although McDonald [4] and McDonald et al. [15] included most of the basal hadrosauroids, the results were poorly resolved and most taxa were clustered in a polytomy in the strict consensus tree. Thus, the phylogeny presented in [4] is based on a maximum agreement subtree that entailed the deletion of 30 operational taxonomic unites, while that of [15] is based on Adams consensus tree.

In order to assess the phylogenetic position of the new taxon, we performed a cladistic analysis based on Sues and Averianov [6]. This data matrix has been used by one of the authors (YHL) on the study of *Jintasaurus* [16]. In the current analysis *Nanyangosaurus*, *Shuangmiaosaurus*, and *Yunganglong* were added to the matrix, to increase its taxonomic representation. This new data matrix was compiled in Mesquite 2.72 [17], and consists of 138 characters and 38 ornithopod taxa (Supporting Information S1). Two character codings were modified: character 37 (Postorbital, length of squamosal process; 0: short, postorbital-squamosal joint reaches point at mid-length of supratemporal fenestra; 1: long, postorbital-squamosal joint near level of posterior border of supratemporal fenestra) is changed from ‘0’ to ‘?’ for *Protohadros* because the character state is not clear based on the original descriptive paper (i. e., the squamosal process of the postorbital is partially present) [9] and a new character state (coronoid process orientation: 2, inclined caudally) is added to reflect this unique feature in *Shuangmiaosaurus* [5]. The data matrix was analyzed by a Traditional search in TNT 1.1 [18] with 100 replications of Wagner trees and tree bisection reconnection (TBR) branch swapping (with 100 trees to save per replicate). All characters were unordered and equally weighted. *Hypsilophodon* was selected as outgroup, as in the original analysis [6].

## Results

### Systematic Paleontology

Dinosauria Owen, 1842 [19]

Ornithischia Seeley, 1887 [20]

Ornithopoda Marsh, 1881 [21]

Iguanodontia Dollo, 1888 [22] sensu Sereno, 2005 [11]

Ankylopollexia Sereno, 1986 [23] sensu Sereno, 2005 [11]

Styracosterna Sereno, 1986 [23] sensu Sereno, 2005 [11]

Hadrosauriformes Sereno, 1997 [24] sensu Sereno, 1998 [25]

Hadrosauroidea Cope, 1870 [26] sensu Sereno, 2005 [11]

*Yunganglong* gen. nov.

urn:lsid:zoobank.org:act:C0BC17BE-AFA2-46E9-9712-3F7767C31C56

*Yunganglong datongensis* sp. nov.

urn:lsid:zoobank.org:act:A906797B-9B90-4C19-AC96-AD8726A7EDD9

#### Holotype

SXMG V 00001 (Shanxi Museum of Geology, Taiyuan, Shanxi Province, China). Associated but disarticulated partial skeleton of one individual, including the caudodorsal part of the skull (ZY007-37 and -38, separated along the floor of the braincase), two cervical vertebrae (-40 and -41), partial dorsal neural arch and neural processes (-36), two caudals (-27, proximal; -19, middle), distal portions of both ischia (-11, left; -12, right), distal end of left femur (-32), proximal portion of right tibia (-1), and distal portion of left tibia with astragalus (-2). See Table 1 for measurements.

**Table 1:** Measurements of bones of the holotype of *Yunganglong datongensis* (SXMG V 00001) (in millimeters).

Bone	Dimension	Measurement
Caudodorsal part of the skull (ZY007-37, -38)	Width between the pendent paroccipital processes	370
	Height from the apex of the supraoccipital to the ventral edge of the occipital condyle	175
	Length of preserved parietal	140
	Midline length from the caudal end of the parietal to the caudal end of the paroccipital processes	120
Cervical (ZY007-40)	Length of centrum including cranial articular process	135
	Length of centrum	90
	Width of cranial articular surface	105
	Width of caudal articular surface	140
	Height of centrum on the cranial articular surface	88
	Height of total vertebra	195
	Diameter of neural canal	36
Proximal caudal (ZY007-27)	Length of centrum	89
	Width of cranial articular surface	160
	Width of caudal articular surface	156
	Height of centrum on the cranial articular surface	175
	Height of total vertebra	540
	Diameter of neural canal	23
Middle caudal (ZY007-19)	Length of centrum	95
	Width of cranial articular surface	107
	Width of caudal articular surface	110
	Height of centrum on the cranial articular surface	123
	Height of total vertebra	330
	Diameter of neural canal	23
Distal portion of left ischium (ZY007-11)	Length	295
	Cross section at proximal end (length/width/perimeter)	49/44/160
	Expanded distal end (length/width/perimeter)	100/70/290
Distal portion of right ischium (ZY007-12)	Length	270
	Cross section at proximal end (length/width/perimeter)	50/48/170
	Expanded distal end (length/width/perimeter)	97/69/290
Distal end of left femur (ZY007-32)	Preserved length	320
	Cross section of preserved proximal end (length/width/perimeter)	103/66/490
	Craniocaudal length of medial condyle	270
	Craniocaudal length of lateral condyle	240
Proximal portion of right tibia (ZY007-1)	Preserved length	565
	Cross section of preserved distal end (length/width/perimeter)	110/100/350
Distal portion of left tibia (ZY007-2)	Preserved length	385
	Cross section of preserved distal end (length/width/perimeter)	120/97/360

#### Etymology

The generic name “Yungang” is after “Yungang Grottoes”, a UNESCO World Heritage built in the 5^th^ and 6^th^ centuries about 50 km east of the fossil locality; “long” means “dragon” in Chinese. The specific name reflects “Datong”, the city in which the locality is situated.

#### Locality and horizon

In the vicinity of Zuoyun County, Datong City, Shanxi Province, P. R. China. Lower part of Zhumapu Formation, lower Upper Cretaceous [27].

In the Zuoyun area, the Zhumapu Formation reaches 722 m in thickness. It overlies the Lower Cretaceous Zuoyun Formation with a parallel unconformity relationship and is covered by Tertiary basalts. The age of the Zhumapu Formation is determined by biostratigraphic correlations, including evidences from angiosperm plants, spores and pollens, ostracodes, and bivalves [27]. No radiometric-based age has been obtained for this formation.

#### Differential diagnosis (for genus and species by monotypy)

Basal hadrosauroid with the following unique combination of four character states: 1) caudal surface of the supraoccipital inclined steeply forward at approximately 45^0^ (present in *Yunganglong* and more advanced taxa, but nearly vertical in *Jintasaurus* and less derived Hadrosauriformes); 2) caudolaterally extended horizontal portion of the paroccipital process and accompanying squamosal (present in *Yunganglong* and more advanced taxa, but laterally extended in *Jintasaurus* and less derived Hadrosauriformes); 3) pendent portion of the paroccipital process does not curve cranially (observed in *Yunganglong* and *Jintasaurus*; the pendant portion does curve cranially in *Bactrosaurus* and more advanced taxa); 4) intercondylar extensor groove of the femur deep, U-shaped, partially enclosed by expansion of medial and lateral condyles (present in *Yunganglong* and less derived Hadrosauriformes, but canal fully enclosed by fusion of lateral and medial condyles in *Nanyangosaurus* and more advanced taxa).

### Specimen Description

**Figure 2:**
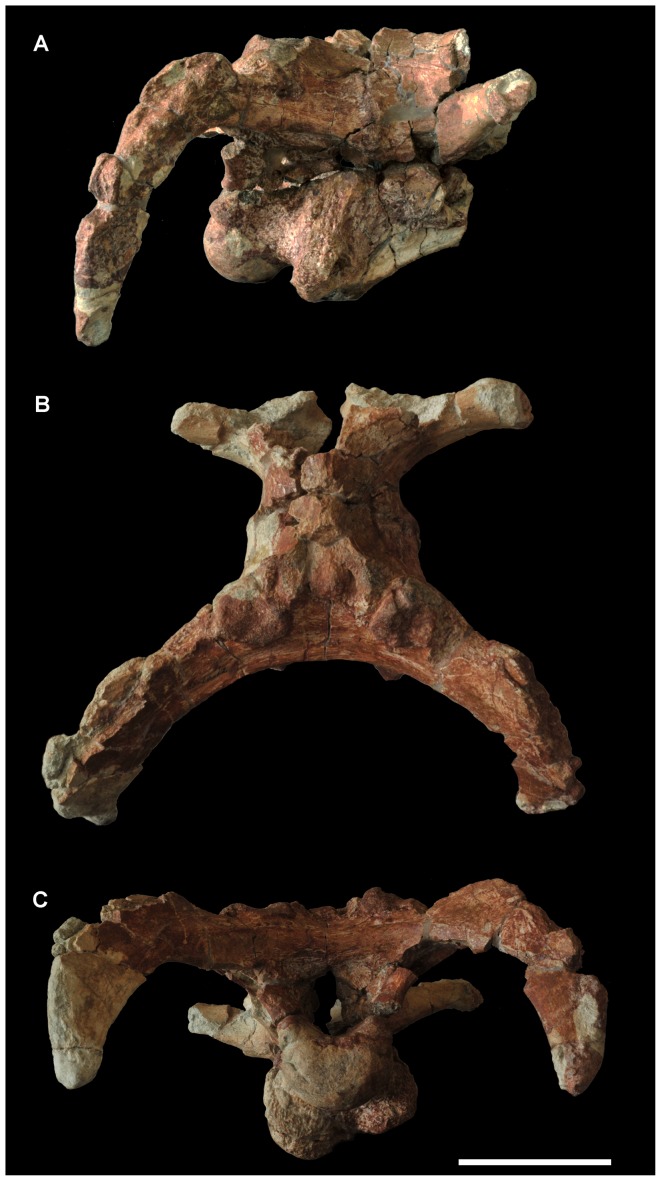
Photos of caudodorsal part of the skull of *Yunganglong datongensis* (SXMG V 00001). (A)-Right lateral view. (B)-Dorsal view. (C)-Caudal view. Scale bar = 10 cm.

**Figure 3:**
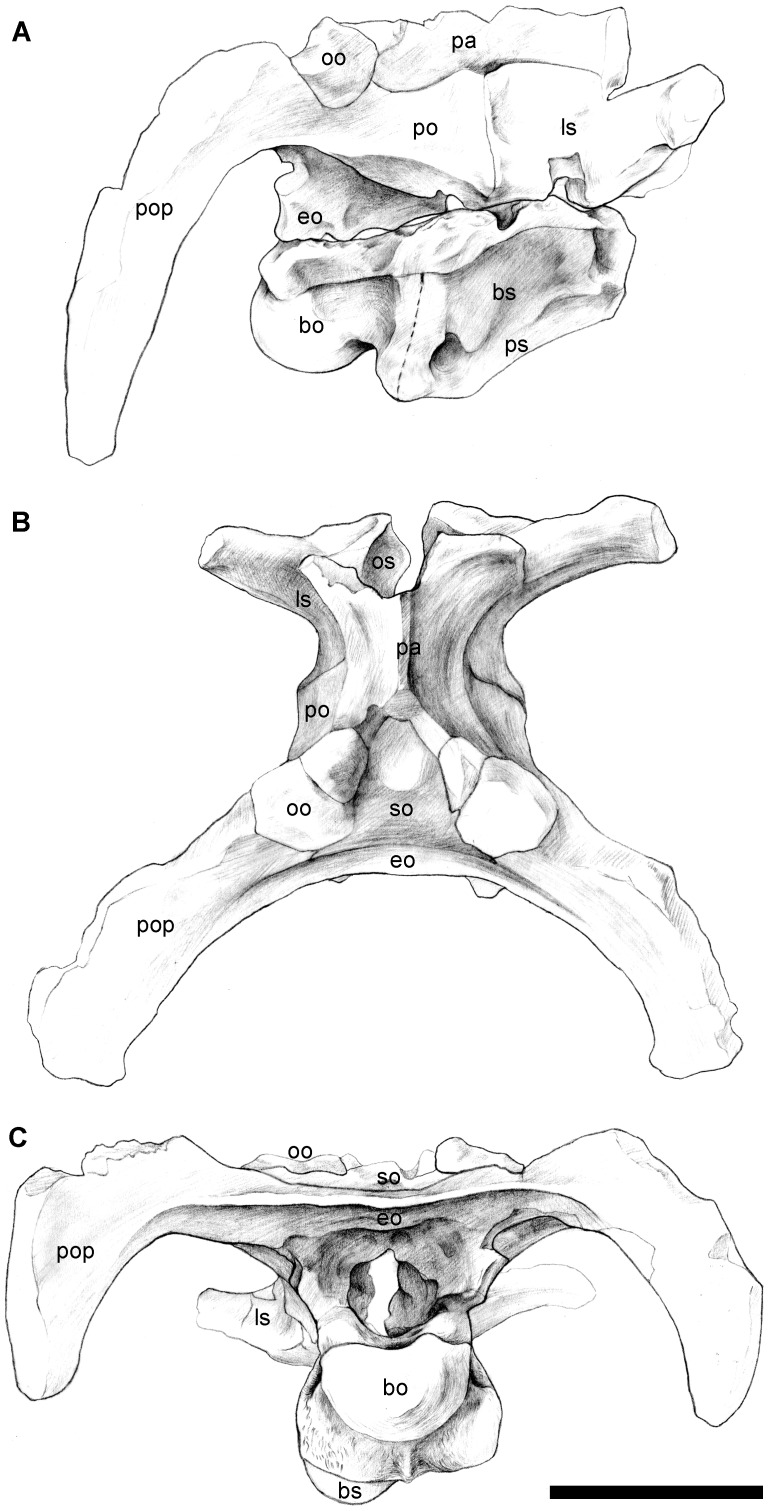
Interpretive drawings of caudodorsal part of the skull of *Yunganglong datongensis* (SXMG V 00001). (A)-Right lateral view. (B)-Dorsal view. (C)-Caudal view. Scale bar = 10 cm. bo = basioccipital, bs = basisphenoid, eo = exoccipital, ls = laterosphenoid, oo = opisthotic, pa = parietal, po = prootic, pop = paroccipital process, ps = parasphenoid, so = supraoccipital, sq = squamosal.

The caudodorsal part of the skull is preserved, including the parietal, paroccipital processes, and the almost complete braincase (Figs. 2, 3). In occipital view, the skull is transversely wide and dorsoventrally low, with the width between the pendent paroccipital processes of 37.0 cm and the height from the apex of the supraoccipital to the ventral edge of the occipital condyle of 17.5 cm.

In dorsal view, the preserved parietal length is 14.0 cm, and the narrowest transverse width at the mid-length of the parietals is 6.0 cm. There is a low sagittal crest along the midline of the preserved parietal portion, but whether or not it continued caudally to form a tall nuchal crest as in *Levnesovia* [6] and *Jintasaurus* [13] is not clear.

The caudal surface of the supraoccipital is steeply inclined rostrally; the bone is nearly horizontal. In occipital view, the dorsal portion of the supraoccipital consists of a median knob-like dorsal process and two dorsolateral projections. Ventrally, the supraoccipital is separated from the exoccipitals by a slit-like transverse groove on the latter, which continues laterally underneath the opisthotics. The ventral edge of this groove forms a strong horizontal ridge, which borders the foramen magnum dorsally. The lateral sides of the foramen magnum are enclosed by the exoccipitals, which bear a knob-like ventral corner on each side. The exoccipitals also meet along the floor of the foramen magnum.

The paroccipital processes strongly bend caudolaterally, enclosing an arc of around 100 degrees in dorsal view and thereby, increasing the length of skull. The longitudinal distance from the caudal end of the parietal to an imaginary point on the midline between the caudal ends of the paroccipital processes is 12.0 cm, almost the same length as the parietal. In lateral view, the straight and vertical pendent portions of the paroccipital processes are set caudal to the occipital condyle, with their ventral tips slightly below the ventral edge of the occipital condyle.

The occipital condyle is formed by the basioccipital and describes a crescent in caudal view. Its caudal surface is vertical and slightly bulging, while its ventral surface is strongly convex. The surface is generally smooth, and one groove is visible on the left half of the caudal surface (Fig. 2C). A short neck divides the occipital condyle and the basal tubera. The basal tubera seem to be formed more by the basisphenoid than by the basioccipital, and those two bones are well fused to each other. The basal tubera are separated by a longitudinal median recess, and a delicate ridge appears along the midline. A round craniolaterally facing fossa exists on the cranial surface of each side of the basal tubera.

The braincase is broken along a line through the exits of the cranial nerves. The positions of these cranial nerve exits are conventional compared to other hadrosauroids, and generally correspond to those in closely related taxa, such as *Levnesovia*. Three foramina pierce the fused opisthotic and exoccipital laterally for cranial nerves X-XII. The rostral two foramina for cranial nerves X and XI are merged, giving a larger appearance than the caudal one for cranial nerve XII. The auditory recess, which includes the fenestra vestibule (fenestra ovalis of [6]), exit for cranial nerve IX, and foramen for the jugular vein, is bounded by the fused basisphenoid and parasphenoid ventrally, the prootic dorsally, and the fused opisthotic and exoccipital caudally. Rostral to the auditory recess is the large, round trigeminal foramen.

**Figure 4:**
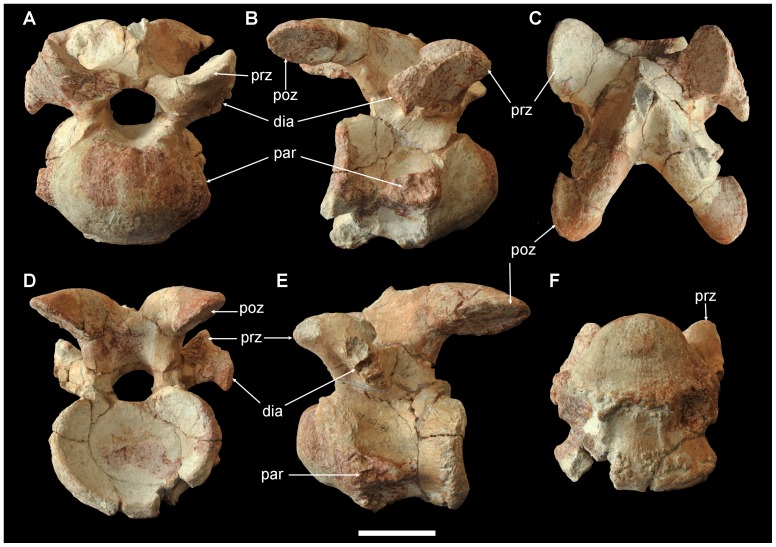
Cervical vertebra of *Yunganglong datongensis* (SXMG V 00001). (A)-Cranial view. (B)-Right lateral view. (C)-Dorsal view. (D)-Caudal view. (E)-Left lateral view. (F)-Ventral view. Abbreviations: dia, diapophysis; par, parapophysis; poz, postzygapophysis; prz, prezygapophysis. Scale bar = 6 cm.

Two cervicals (ZY007-40, -41) are preserved, and ZY007-40 is almost complete (Fig. 4). The centrum is strongly opisthocoelous, with the round cranial articular condyle more than half the length of the centrum. The cranial-most protruding point is above the midlevel of the centrum. The caudal articular surface is deeply concave and elliptical in caudal view, being wider than high. The lateral side of the centrum is slightly higher than long, and is divided by a midlevel ridge into dorsal and ventral depressions. The large parapophysis occupies the cranial half of this ridge. The ventral surface is slightly convex, especially at the caudal half. The neural canal is large and round, about half the height of the centrum. The prezygapophysis starts from the dorsolateral corner of the cranial half of the neural arch and does not protrude beyond the cranial edge of the articular condyle. The dorsomedially facing smooth articular facet is large, oval, and flat. The short stem of the prezygapophysis also gives rise to the diapophysis, which projects caudoventrolaterally. The robust postzygapophysis is directed more caudally than laterally, with its entire articular facet beyond the caudal edge of the centrum. The neural spine is not well preserved, but seems to have been delicate.

**Figure 5:**
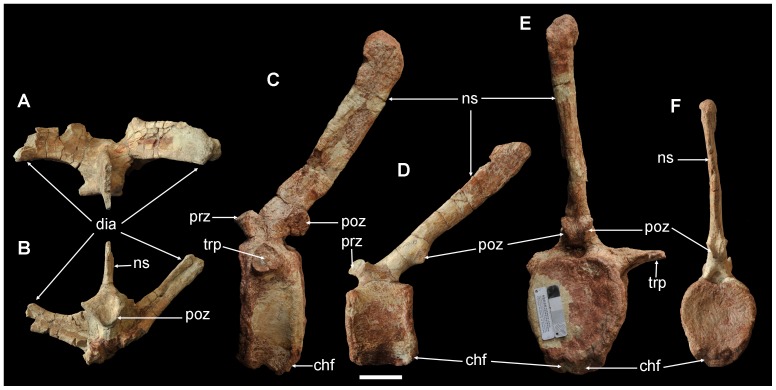
Partial dorsal and two caudal vertebra of *Yunganglong datongensis* (SXMG V 00001). (A, B)-Partial dorsal neural arch and neural spine in (A) dorsal and (B) caudal views. (C, E)-Proximal caudal in (C) left lateral and (E) caudal views. (D, F)-Middle caudal in (D) left lateral and (F) caudal views. Abbreviations: chf, chevron facet; dia, diapophysis; ns, neural spine; poz, postzygapophysis; prz, prezygapophysis; transverse process, trp. Scale bar = 6 cm.

A partial dorsal neural arch and neural spine are preserved (ZY007-36) (Fig. 5A, B). The diapophysis projects dorsolaterally and slightly caudally and has a triangular cross section. The long, oval, and flat postzygapophysis articular facet faces lateroventrally. Only the basal portion of the neural spine is preserved; it is plate-like and thin transversely.

One proximal (ZY007-27) (Fig. 5 C, E) and one middle (ZY007-19) (Fig. 5 D, F) caudal are preserved. The proximal caudal's centrum is narrow and slightly amphicoelous. The articular surfaces are elliptical with relatively narrow ventral ends. The partial right transverse process starts at the neurocentral junction and projects laterally. The neural spine is tall, almost double the height of the centrum. The cross section of the neural spine is rectangular, with the transverse width less than half the longitudinal length. The tip of the neural spine expands slightly. The centrum of the middle caudal is dorsoventrally lower but proximodistally longer than that of the proximal caudal, with hexagonal articular surfaces. The transverse process is not present, and the neural spine projects caudodorsally.

**Figure 6:**
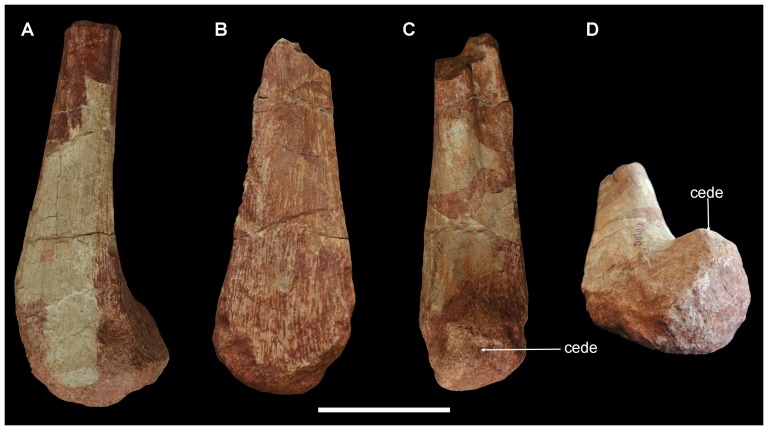
Distal portions of ischia of *Yunganglong datongensis* (SXMG V 00001). (A, C)-Distal portion of left ischium in (A) medial and (C) cranial views. (B, D)-Distal portion of right ischium in (C) medial and slightly caudal and (D) distal and slightly dorsal views. Abbreviation: cede, cranially expanded distal end. Scale bar = 10 cm.

The distal portions of both ischia are preserved (ZY007-11 left, -12 right) (Fig. 6). The preserved shaft is triangular in cross section, with a flat medial surface to contact its counterpart. This flat surface continues to the distal end. The craniolateral surface bears a shallow longitudinal depression, while the caudolateral surface is flat. The distal end slightly expands craniolaterally to form a hemispherical expansion. The expanded distal end is about double the width of the shaft diameter.

**Figure 7:**
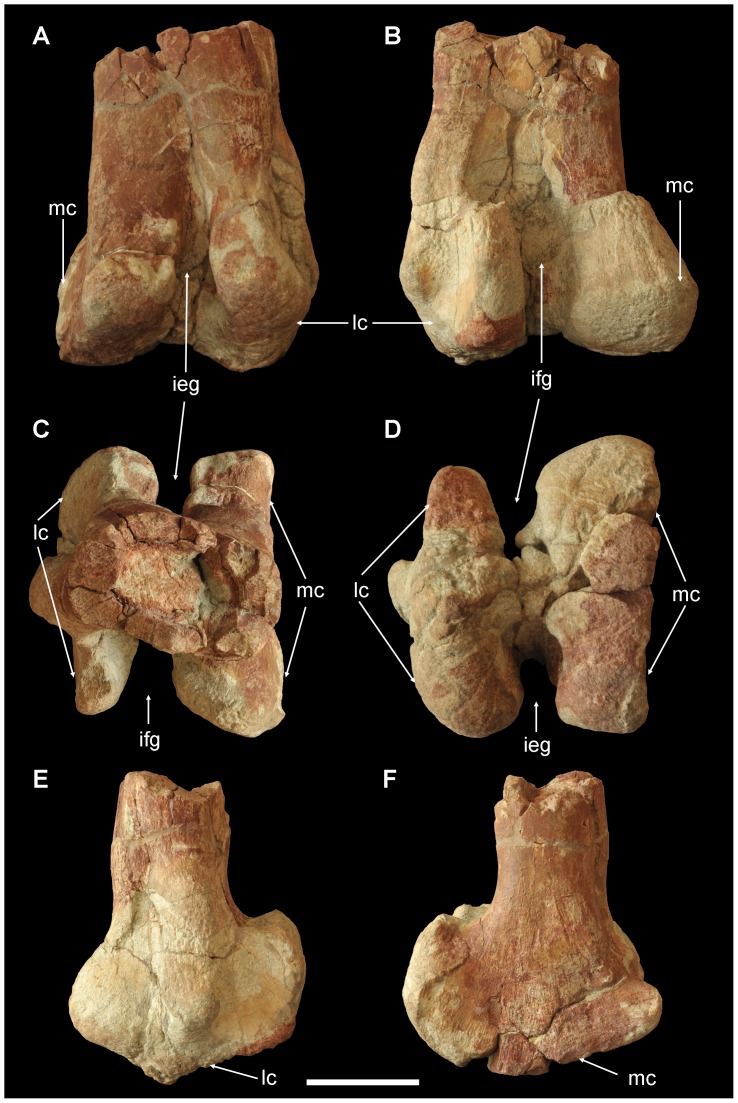
Distal end of left femur of *Yunganglong datongensis* (SXMG V 00001). (A)-Cranial view. (B)- Caudal view. (C)-Proximal view. (D)-Distal view (E)-Lateral view. (F)-Medial view. Abbreviations: ieg, intercondylar extensor groove; ifg, intercondylar flexor groove; lc, lateral condyle; mc, medial condyle. Scale bar = 10 cm.

The distal end of the left femur is preserved (ZY007-32). The cross section of the preserved proximal end of the shaft is elliptical, and its transverse width is twice its craniocaudal width. The intercondylar extensor groove of the femur is deep, U-shaped, partially enclosed by expansion of medial and lateral condyles. The medial condyle is slightly larger than the lateral. The medial surface of the medial condyle is flat, while the lateral surface of the lateral condyle bears a stout longitudinal ridge. The caudal condyles are strongly developed, fan-shaped, and longer proximodistally than craniocaudally in lateral and medial views. In caudal view, the medial condyle is more expanded than the lateral, about three times wider transversely. On the lateral surface of the medial condyle, a small longitudinal ridge develops close to the shaft (Fig. 7D).

**Figure 8:**
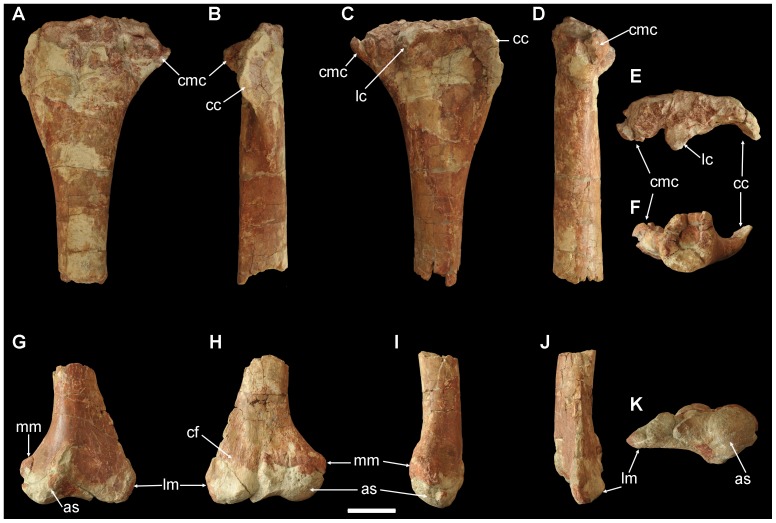
Tibia and astragalus of *Yunganglong datongensis* (SXMG V 00001). (A–F)-Proximal portion of right tibia in (A) media, (B) cranial, (C) lateral, (D) caudal, (E) proximal, and (F) distal views. (G–K)-Distal portion of left tibia with astragalus in (G) cranial, (H) caudal, (I) medial, (J) lateral, and (K) distal views. Abbreviations: as, astragalus; cc, cnemial crest; cf, concavity for articulation of distal end of fibula; cmc, caudomedial condyle; lc, lateral condyle; lm, lateral malleolus; mm, medial malleolus. Scale bar = 10 cm.

The proximal portion of the right tibia (ZY007-1) (Fig. 8 A–F) and the distal portion of the left tibia with astragalus (ZY007-2) (Fig. 8 G–K) are preserved. The cnemial crest is well developed and protrudes craniolaterally. Two laterally-projecting condyles exist on the caudal half of the proximal end. The midshaft is ovate in cross section. The distal end of the left tibia is articulated with the astragalus. In cranial view, the astragalus is triangular, with a clear short ascending process. In caudal view, the astragalus is also triangular, with a delicate ascending process at its proximolateral corner.

### Phylogenetic Analysis

**Figure 9:**
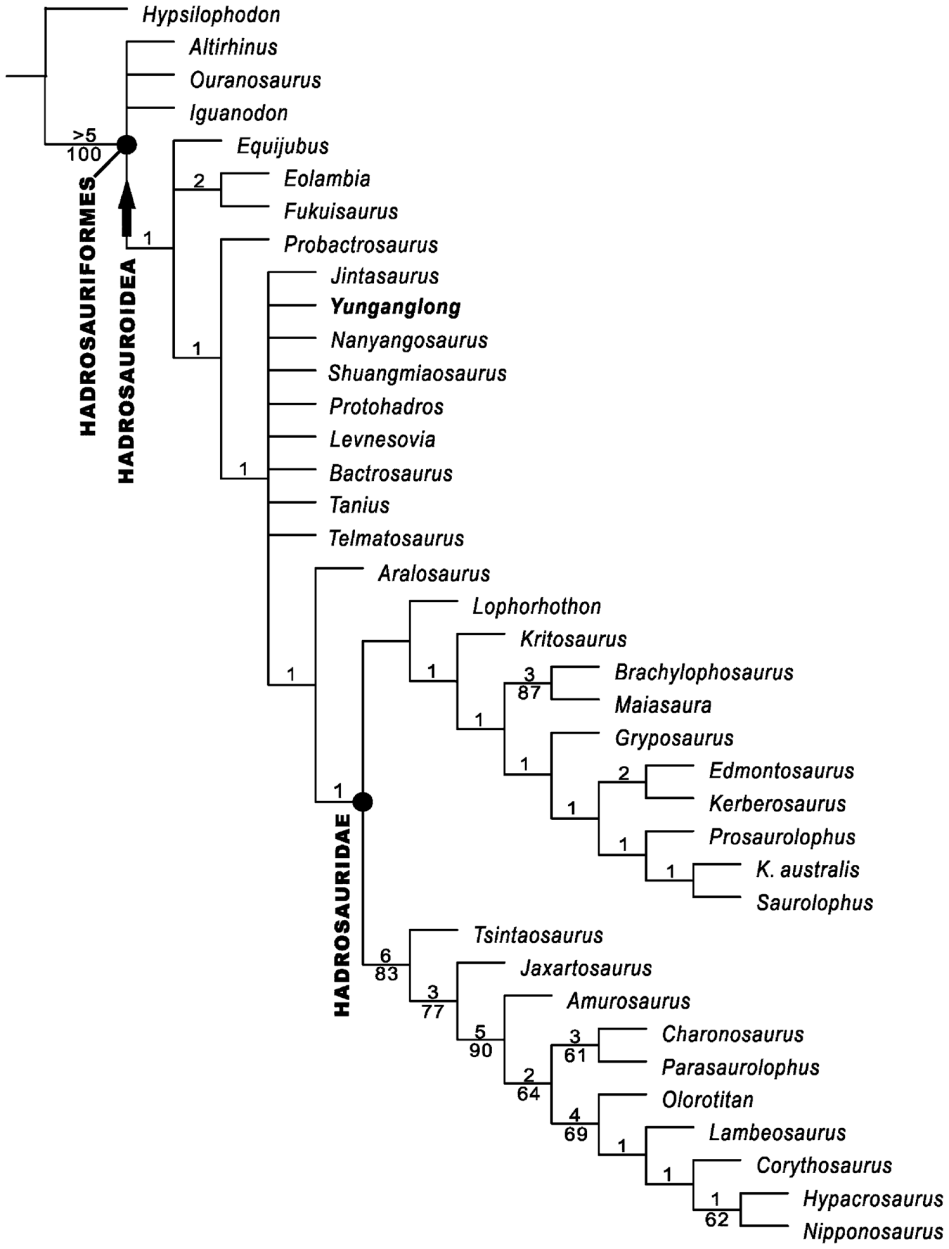
Phylogenetic relationships of *Yunganglong datongensis* (SXMG V 00001) based on strict consensus tree obtained in this study. Bremer supports no less than 1 and bootstrap frequencies more than 50 are indicated above and below relevant branches, respectively. “•” represents node-based definition, and “→” represents branch-based definition.

Phylogenetic analysis recovered 303 MPTs (most parsimonious trees) of 294 steps, with a CI (consistency index) of 0.534 and a RI (retention index) of 0.858. The strict consensus tree recovered *Yunganglong*, *Jintasaurus*, *Protohadros*, *Nanyangosaurus*, *Shuangmiaosaurus*, *Levnesovia*, *Bactrosaurus*, *Tanius*, and *Telmatosaurus* in an unresolved polytomy between *Probactrosaurus* and (*Aralosaurus*+Hadrosauridae) with poor bootstrap frequencies and bremer supports (Fig. 9).

In the strict consensus tree, the Hadrosauroidea clade is supported by 7 unambiguous synapomorphies (40[1], parietal sagittal crest long, more than 2/3 length of parietal; 46[1], antorbital fenestra external opening absent; 57[1], lacrimal-nasal contact absent; 74[1], length of basipterygoid processes extending well below level of ventral border of occipital condyle; 75[1], occipital condyle articular surface vertical; 103[1], cervical zygapophyseal peduncles on arches elevated, extending well above level of neural canal, zygapophyses long and dorsally arched; 114[1], humeral distal condyles compressed mediolaterally, flaring little from shaft of humerus), the (*Probactrosaurus*+more advanced taxa) clade is supported by 4 unambiguous synapomorphies (55[1], line of alveolar foramina on medial side of maxilla located dorsal to mid-height of maxilla; 83[1], dentary alveolar trough narrow parallel-sided grooves; 94[1], at least 2, and often 3, teeth in vertical series contributing to occlusal surface per tooth position; 96[1], ornamentation on labial surface of maxillary teeth absence of all but primary carina), the clade above *Probactrosaurus* is strongly supported by 19 unambiguous synapomorphies (37[1], squamosal process of postorbital long, postorbital-squamosal joint near level of posterior border of supratemporal fenestra; 38[0], parietal long, length/minimum width ratio greater than 2; 51[1], jugal process of maxilla reduced to short projection but retaining a distinct facet; 52[1], location of apex at or anterior to center of maxilla in lateral exposure; 56[1], ectopterygoid-jugal contact absent, palatine-jugal contact enhanced; 65[1], quadratojugal foramen absent; 66[1], well-defined paraquadrate notch of quadrate absent, reduced to poorly defined embayment of quadrate; 72[1], paroccipital process and accompanying squamosal curved anteriorly; 77[1], predentary gracile and shovel-shaped, straight to gently rounded anterior margin, numerous nutrient foramina across entire anterior margin, rounded, triangular denticles project anteriorly and fit into a continuous transverse slot on underside of premaxilla; 89[1], surangular foramen absent; 93[1], 3 or more replacement teeth per tooth position; 99[1], dentary tooth crown dominated by on primary ridge on lingual surface, secondary ridges faint if present; 101[1], 12–13 cervical vertebrae; 106[1], scapular proximal end dorsoventrally narrow, no wider than distal portion of scapula, acromion process projects horizontally, anteroventral corner notched, articulation restricted; 118[2], manual digit I absent; 128[1], pubis obturator foramen fully open, tubercle absent; 131[1], femoral distal condyles expanded anteriorly, with anterior ends meeting or fusing together and enclosing extensor tunnel; 132[1], tibia cnemial crest extending on diaphysis; 137[1], pedal unguals dorsoventrally flattened and broadened, hoof-like), the (*Aralosaurus*+Hadrosauridae) clade is supported by 8 unambiguous synapomorphies (41[1], squamosals approaching midline, separated by narrow band of parietal; 54[1], ectopterygoid ridge of maxilla well developed into lateral and well demarcated border continuous along posterior region of maxilla; 58[1], jugal expanded dorsoventrally in front of orbit, lacrimal pushed dorsally to lie completely above the level of the maxilla, jugal forms lower portion of orbital rim; 60[1], free ventral flange of jugal present, jugal dorsoventrally constricted beneath infratemporal fenestra to set off flange anterior to constriction; 61[1], ventral flange of jugal rounded or lobate; 62[1], ratio of depth of jugal at constriction below infratemporal fenestra to length of free flange on jugal small, 0.70–0.90; 95[1], maxillary tooth crown elongate and lanceolate, height/width ratio at least 2.5 at center of tooth row; 98[1], apex of dentary teeth central, tooth straight and nearly symmetrical), and the Hadrosauridae clade is supported by 5 unambiguous synapomorphies (45[1], posteroventral surface of squamosal forming deep, nearly vertical, well-exposed face in posterior view; 63[1], jugal contributed to infratemporal fenestra, acute angle between postorbital bar and jugular bar; 71[1], ventrolateral corner of supraoccipital inset into exoccipital so that supraoccipital is “locked” between exoccipitals; 92[1], greater than 32 tooth positions in dentary and maxillary tooth rows; 112[1], deltopectoral crest of humerus extends at least to midshaft or longer) (Supporting Information S2).

The 50% majority rule tree further resolved (*Yunganglong*, *Jintasaurus*, *Protohadros*, *Nanyangosaurus*, and *Shuangmiaosaurus*) as more basal than a trichotomy of *Tanius*, (*Levnesovia*+*Bactrosaurus*), and (*Telmatosaurus*+more advanced taxa). Mapping synapomorphies onto the majority rule tree showed that the clade (*Yunganglong*, *Jintasaurus*, *Protohadros*, *Nanyangosaurus*, *Shuangmiaosaurus*+more advanced taxa) is supported by 9 unambiguous synapomorphies (37[1], squamosal process of postorbital long, postorbital-squamosal joint near level of posterior border of supratemporal fenestra; 38[0], parietal long, length/minimum width ratio greater than 2; 51[1], jugal process of maxilla reduced to short projection but retaining a distinct facet; 65[1], quadratojugal foramen absent; 66[1], well-defined paraquadrate notch of quadrate absent, reduced to poorly defined embayment of quadrate; 93[1], 3 or more replacement teeth per tooth position; 118[2], manual digit I absent; 132[1], tibia cnemial crest extending on diaphysis; 137[1], pedal unguals dorsoventrally flattened and broadened, hoof-like), while the clade ((*Levnesovia*+*Bactrosaurus*)+*Tanius*+(*Telmatosaurus*+more advanced taxa)) is supported by 8 unambiguous synapomorphies (4[1], present of premaxillary foramen ventral to anterior margin of external nares that opens onto the palate and connected by a canal to the narial fossa; 52[1], location of apex at or anterior to center of maxilla in lateral exposure; 56[1], ectopterygoid-jugal contact absent, palatine-jugal contact enhanced; 72[1], paroccipital process and accompanying squamosal curved anteriorly; 77[1], predentary gracile and shovel-shaped, straight to gently rounded anterior margin, numerous nutrient foramina across entire anterior margin, rounded, triangular denticles project anteriorly and fit into a continuous transverse slot on underside of premaxilla; 89[1], surangular foramen absent; 99[1], dentary tooth crown dominated by on primary ridge on lingual surface, secondary ridges faint if present; 129[1], shaft of ischium nearly straight in lateral view) (Supporting Information S3).

The strict consensus tree recovered by our analysis is more resolved than those consensus trees of [4] and [15] regarding the phylogeny of basal hadrosauroids, although the relationships among *Yunganglong*, *Jintasaurus*, *Protohadros*, *Nanyangosaurus*, *Shuangmiaosaurus*, *Levnesovia*, *Bactrosaurus*, *Tanius*, and *Telmatosaurus* are still not resolved. The single most parsimonious tree of [13] recovered *Protohadros*, *Tanius*, *Bactrosaurus*, *Gilmoreosaurus*, and *Telmatosaurus* as successively more derived than *Probactrosaurus*; in contrast, in our analysis, although *Probactrosaurus* is recovered as more basal than *Protohadros*, *Tanius*, *Bactrosaurus*, and *Telmatosaurus*, the relationships among the latter four plus *Yunganglong*, *Jintasaurus*, *Nanyangosaurus*, *Shuangmiaosaurus*, *Levnesovia* are not resolved. The analysis of [14] focused on saurolophine hadrosauroids and only used two basal hadrosauroids, with *Probactrosaurus* more basal than *Bactrosaurus*.

## Comparison and Discussion

In *Yunganglong* the caudal surface of the supraoccipital is inclined steeply forward at approximately 45^0^. In contrast, the caudal surface is nearly vertical in *Jintasaurus* and less derived Hadrosauriformes. The condition in *Protohadros*, *Nanyangosaurus*, and *Shuangmiaosaurus* is not known.

*Yunganglong* has relatively poorly developed cranial expansion of the distal condyles of the femur and a moderately deep and fully opened intercondylar extensor groove. In contrast, in *Nanyangosaurus*, *Bactrosaurus*, *Levnesovia*, *Tanius*, *Telmatosaurus*, and Hadrosauridae, the cranial ends of the expanded condyles meet or fuse together and enclose the extensor tunnel. The condition in *Jintasaurus*, *Protohadros*, and *Shuangmiaosaurus* is not known.

*Nanyangosaurus* is a medium-sized (∼4.5 m) basal hadrosauroid represented by a nearly complete post-cervical vertebral column, the distal portions of both ischia, and elements of the fore and hind limbs. The age of the *Nanyangosaurus*-bearing red beds in the Xiaguan Basin of southern Henan Province was argued to be Early Cretaceous by Xu et al. [7] based on the evolutionary grade of *Nanyangosaurus*, which is intermediate between *Probactrosaurus* and Hadrosauridae. However, the data matrix of Xu [7] only included seven hadrosauroids (plus *Hypsilophodon*, *Camptosaurus*, *Iguanodon*, and *Ouranosaurus*), and our results and those of another cladistic analysis [4] suggest a more derived position for *Nanyangosaurus*. Furthermore, regional geological workers insist on an early Late Cretaceous age for the *Nanyangosaurus*-bearing red beds in Xiaguan Basin of southern Henan Province, which is partially equivalent to the famous dinosaur egg-bearing red beds in the neighboring Xixia Basin [28], [29]. They also prefer the use of the name Xiaguan Formation to refer to the red beds in the Xiaguan Basin based on priority [29]. Therefore, we regard the horizon of *Nanyangosaurus* to be the Xiaguan Formation of early Late Cretaceous age.

*Shuangmiaosaurus* was recovered from the Late Cretaceous of Liaoning Province of northeastern China, and is represented by a left maxilla (holotype) and a referred left dentary [5]. The lack of overlapping elements between it and *Yunganglong* precludes further comparison.

The available information does not allow direct anatomical comparisons between *Yunganglong* and two other early Late Cretaceous non-hadrosaurids from North America: the Cenomanian *Protohadros* [9] and the Turonian *Jeyawati* [10]. The North American Campanian *Glishades* is probably an indeterminate member of Hadrosauridae [30] rather than a non-hadrosaurid hadrosauroid [31]. The late Campanian - early Maastrichtian European *Tethyshadros* is an insular dwarf with a possible close relationship to *Telmatosaurus* [32].

*Eolambia caroljonesa* from the lower Cenomanian Mussentuchit Member of the Cedar Mountain Formation in eastern Utah recently received a detailed redescription and was recovered as the sister taxon of *Probactrosaurus gobiensis* from the Early Cretaceous of China [30]. *Eolambia* is different from *Yunganglong* in numerous features, such as the nearly vertical caudal surface of the supraoccipital, the rostrally curved distal end of the paroccipital process, the caudoventrally-directed occipital condyle, and the expanded boot-like distal end of the ischium. All these features are absent in *Yunganglong*.

Young [12] assigned the hadrosauroid material recovered from the Zuoyun area to *Bactrosaurus johnsoni*. Unfortunately, no overlapping material can be compared between Young's material and *Yunganglong*. However, based on the more basal phylogenetic position and lower stratigraphic horizon of *Yunganglong* compared to *Bactrosaurus johnsoni*, Young's material probably does not belong to *Bactrosaurus* but might pertain to *Yunganglong*.

## Supporting Information

Supporting Information S1 **Data Matrix.tnt.**
(TNT)

Supporting Information S2 **Synapomorphies of strict consensus.**
(EMF)

Supporting Information S3 **Synapomorphies of 50 majority.**
(EMF)
